# PTP1B Inhibitory and Anti-Inflammatory Effects of Secondary Metabolites Isolated from the Marine-Derived Fungus *Penicillium* sp. JF-55

**DOI:** 10.3390/md11041409

**Published:** 2013-04-23

**Authors:** Dong-Sung Lee, Jae-Hyuk Jang, Wonmin Ko, Kyoung-Su Kim, Jae Hak Sohn, Myeong-Suk Kang, Jong Seog Ahn, Youn-Chul Kim, Hyuncheol Oh

**Affiliations:** 1 Hanbang Body-Fluid Research Center, Wonkwang University, Iksan 570-749, Korea; E-Mail: hsds@wku.ac.kr; 2 Chemical Biology Research Center, Korea Research Institute of Bioscience and Biotechnology (KRIBB), 30 Yeongudanji-ro, Ochang, Cheongwon 363-883, Korea; E-Mails: jangjh@kribb.re.kr (J.-H.J.); jsahn@kribb.re.kr (J.S.A.); 3 College of Pharmacy, Wonkwang University, Iksan 570-749, Korea; E-Mails: rabis@wku.ac.kr (W.K.); pipo5@wku.ac.kr (K.-S.K.); 4 Standardized Material Bank for New Botanical Drugs, College of Pharmacy, Wonkwang University, Iksan 570-749, Korea; 5 College of Medical and Life Sciences, Silla University, Busan 617-736, Korea; E-Mails: jhsohn@silla.ac.kr (J.H.S.); shinhwa9575@nate.com (M.-S.K.)

**Keywords:** *Penicillium* sp., marine-derived fungi, PTP1B inhibitors, anti-inflammatory effect, heme oxygenase-1

## Abstract

Protein tyrosine phosphatase 1B (PTP1B) plays a major role in the negative regulation of insulin signaling, and is thus considered as an attractive therapeutic target for the treatment of diabetes. Bioassay-guided investigation of the methylethylketone extract of marine-derived fungus *Penicillium* sp. JF-55 cultures afforded a new PTP1B inhibitory styrylpyrone-type metabolite named penstyrylpyrone (**1**), and two known metabolites, anhydrofulvic acid (**2**) and citromycetin (**3**). Compounds **1** and **2** inhibited PTP1B activity in a dose-dependent manner, and kinetic analyses of PTP1B inhibition suggested that these compounds inhibited PTP1B activity in a competitive manner. In an effort to gain more biological potential of the isolated compounds, the anti-inflammatory effects of compounds **1**–**3** were also evaluated. Among the tested compounds, only compound **1** inhibited the production of NO and PGE_2_, due to the inhibition of the expression of iNOS and COX-2. Penstyrylpyrone (**1**) also reduced TNF-α and IL-1β production, and these anti-inflammatory effects were shown to be correlated with the suppression of the phosphorylation and degradation of IκB-α, NF-κB nuclear translocation, and NF-κB DNA binding activity. In addition, using inhibitor tin protoporphyrin (SnPP), an inhibitor of HO-1, it was verified that the inhibitory effects of penstyrylpyrone (**1**) on the pro-inflammatory mediators and NF-κB DNA binding activity were associated with the HO-1 expression. Therefore, these results suggest that penstyrylpyrone (**1**) suppresses PTP1B activity, as well as the production of pro-inflammatory mediators via NF-κB pathway, through expression of anti-inflammatory HO-1.

## 1. Introduction

Recent studies of marine natural products have focused on marine microorganisms as an untapped source of secondary metabolites [1,2]. Marine microorganisms, particularly marine-derived fungi, are fertile producers of new structurally interesting compounds, and are recognized as an important source of structurally novel and bioactive secondary metabolites for drug discovery [3,4,5,6]. In this respect, we have recently initiated our studies of the secondary metabolites from marine-derived fungi with interest in new pharmacological activities and mechanisms of the activities related to anti-diabetic activity via PTP1B inhibition and anti-inflammatory activity involving heme oxygenase (HO)-1 expression [7,8].

Several protein tyrosine phosphatases (PTPs) play a critical role in the regulation of a variety of cellular processes, such as growth, proliferation and differentiation, metabolism, immune response, cell-cell adhesion, and cell-matrix contacts [9,10]. Protein tyrosine phosphatase 1B (PTP1B) is a major nontransmembrane phosphotyrosine phosphatase found in human tissues, and is a known negative regulator of the insulin-stimulated signal transduction pathway [11]. A number of genetic and biochemical studies have demonstrated that PTP1B is a major negative regulator of insulin receptor signaling [12,13]. In addition, recent studies have shown that the leptin signaling pathway may be attenuated by PTPs, involving PTP1B [13,14]. Taken together, PTP1B is an attractive target in the development of new treatments for type 2 diabetes and other related metabolic syndromes [14,15].

The transcription factor NF-κB has been implicated in the regulation of many genes that encode for mediators of immune, acute-phase, and inflammatory responses. The regulation of NF-κB signaling by HO-1, an enzyme that is essential for heme degradation, is one of the important mechanisms for cellular pathophysiological conditions of inflammation [16,17,18,19,20,21,22,23]. When HO-1 catabolizes heme, three products that can mediate anti-inflammatory effects are released: carbon monoxide (CO), biliverdin, and Fe^2+^. The anti-inflammatory effects of HO-1 and its products, are mediated by inhibiting the production of pro-inflammatory cytokines and chemokines, such as tumor necrosis factor (TNF)-α, interleukin (IL)-1β, and IL-6, in activated macrophages [18,19], as well as reduction in the expression of pro-inflammatory inducible nitric oxide synthase (iNOS), iNOS-induced nitric oxide (NO) production, expression of cyclooxygenase (COX)-2, and COX-2-induced prostaglandin E2 (PGE_2_) production [20,21]. In addition, the redox-dependent transcription factors such as nuclear transcription factor-E2-related factor 2 (Nrf2), which is a master regulator of the anti-oxidant response and NF-κB signaling, have been shown to mediate HO-1 induction. The HO-1 induction is primarily regulated at the transcriptional level, and its induction involves the Nrf2 [22,23].

As a part of our ongoing studies on bioactive secondary metabolites from marine microorganisms in Korea, we investigated the chemical constituents of crude extracts obtained from cultures of the marine-derived fungus *Penicillium* sp. JF-55, leading to the isolation of a new styrylpyrone-type metabolite named penstyrylpyrone (**1**), and two known metabolites anhydrofulvic acid (**2**) and citromycetin (**3**). This study describes the isolation and structure elucidation of these compounds, and their PTP1B inhibitory and anti-inflammatory effects.

## 2. Results and Discussion

### 2.1. Chemical Structures of Compounds **1**–**3** Isolated from the Marine-Derived Fungus *Penicillium* sp. JF-55

**Figure 1:**
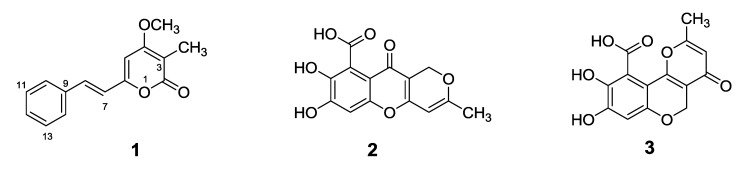
Chemical structures of penstyrylpyrone (**1**), anhydrofulvic acid (**2**) and citromycetin (**3**).

Penstyrylpyrone (**1**) (Figure 1) has the molecular formula of C_15_H_14_O_3_, as deduced from ^13^C NMR (Table 1) and HRESIMS data. This formula indicated nine degrees of unsaturation. The ^13^C NMR spectrum of compound **1** contained only 13 resonances, which implied doubling of two of the resonances. Integration of the resonances in the ^1^H NMR spectrum of compound **1** showed the presence of eight aromatic/olefinic protons. The signals at δ 7.04 (d, 1H, *J* = 16.1 Hz) and δ 7.34 (1H, d, *J* = 16.1 Hz) were attributed to a *trans* double bond unit, and the presence of the signals at δ 7.65 (2H, d, *J* = 7.3 Hz), δ 7.42 (2H, t, *J* = 7.3 Hz), and δ 7.36 (1H, t, *J* = 7.3 Hz) was suggestive of the presence of a phenyl group. The ^1^H NMR data also revealed the presence of a methyl and a methoxy group (Table 1). In addition, ^13^C NMR and DEPT spectra showed signals corresponding to three oxygenated sp^2^ olefins and/or carbonyl carbons, along with two upfield-shifted sp^2^ carbon signals (one methine and one quaternary), and this was suggestive of the presence of a β-oxygenated α-pyrone unit [24]. These units accounted for all degrees of unsaturation. Analysis of 2D NMR data such as HSQC and HMBC data (Table 1), along with a comparison of the shift data with the data of related compounds [24,25,26,27] resulted in the identification of an α-pyrone unit and its substitution pattern to afford the complete structure of compound **1** as shown. The other isolated compounds were identified as (**2**) anhydrofulvic acid [28] and (**3**) citromycetin [29] (Figure 1) by comparing their NMR and MS data with data in previously published literature values.

**Table 1:** NMR spectroscopic data for penstyrylpyrone (**1**) in DMSO-*d*_6_.

Position	δ_H_ (int, mult., *J* in Hz) ^a^	δ_C_^b^	HMBC (H → C)
2	--	163.4	--
3	--	101.0	--
4	--	165.8	--
5	6.76 (1H, s)	97.2	3, 4, 6, 7
6	--	156.7	--
7	7.04 (1H, d, 16.1)	119.9	5, 6, 9
8	7.34 (1H, d, 16.1)	133.4	6, 9, 14
9	--	135.2	--
10/14	7.65 (2H, d, 7.3)	127.3	8, 12, 14
11/13	7.42 (2H, t, 7.3)	128.9	9, 12, 14
12	7.36 (1H, t, 7.3)	129.2	14
15	1.83 (3H, s)	8.8	2, 3, 4
4-OCH_3_	3.93 (3H, s)	56.7	4

^a^ Recorded at 400 MHz;^b^ Recorded at 100 MHz.

### 2.2. PTP1B Inhibitory Effects of Compounds **1**–**3**

**Figure 2:**
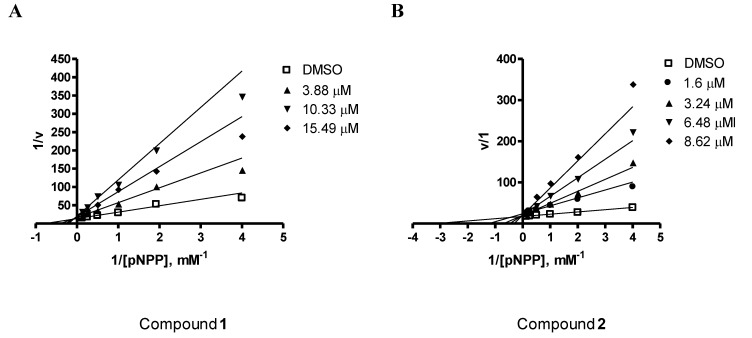
A Lineweaver-Burk plot for penstytrlpyrone (**1**) (**A**) and anhydrofulvic acid (**2**) (**B**) in the inhibition of PTP1B. The data represent the mean values ± SD of three experiments. The concentrations (μM) of penstytrlpyrone (**1**) (**A**) and anhydrofulvic acid (**2**) (**B**) are indicated.

Compounds **1**-**3** (Figure 1) were evaluated for their inhibitory effects against PTP1B activity *in vitro*. Among the tested compounds, penstyrylpyrone (**1**) and anhydrofulvic acid (**2**) exhibited PTP1B inhibitory activity in a dose-dependent manner with IC_50_ values of 5.28 μM and 1.90 μM (Table 2), respectively, while citromycetin (**3**) did not show any inhibitory activity up to 25.8 μM. A known phosphatase inhibitor, ursolic acid (IC_50_ = 3.10 μM) was used as a positive control in the assay [30,31]. The characteristics of PTP1B inhibition by compounds **1** and **2** were then analyzed. PTP1B was incubated with increasing concentrations of the test compounds and full velocity curves were determined (Figure 2) as described in the Experimental section. Non-linear regression analysis showed that the data best fit a competitive model of inhibition, and re-plotting of the data as Lineweaver-Burk transformations confirmed this result, displaying the characteristic intersecting line pattern for competitive inhibition. Thus, penstyrylpyrone (**1**) and anhydrofulvic acid (**2**) were suggested to bind to the active site within PTP1B. To the best of our knowledge, PTP1B inhibitory activity of styrylpyrones and anhydrofulvic acid has not been previously reported. Although compounds **2** and **3** share close structural features, only anhydrofulvic acid (**2**) displayed significant PTP1B inhibitory effects in our assay system, and this finding suggested that the linear tricyclic system and the position of the carbonyl groups in compound **2** might be the important structure features for its binding to the active site of PTP1B.

**Table 2:** PTP1B inhibitory activity with IC_50_ values of compounds **1**–**3**.

Compounds	IC_50_
Ursolic acid	3.10 μM ^a^
Penstyrylpyrone (**1**)	5.28 μM
Anhydrofulvic acid (**2**)	1.90 μM
Citromycetin (**3**)	>25.8 μM

^a^ Positive control.

### 2.3. Effects of Compounds **1**–**3** on the Expression of Pro-Inflammatory Proteins and Production of Pro-Inflammatory Cytokines in Murine Peritoneal Macrophages Stimulated with Lipopolysaccharides (LPS)

**Figure 3:**
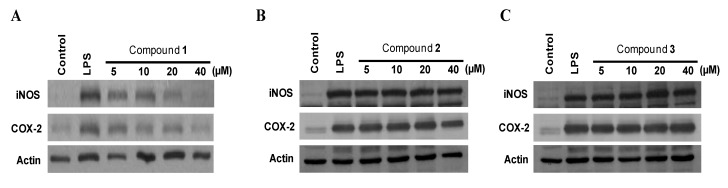
The effects of penstyrylpyrone (**1**) (**A**), anhydrofulvic acid (**2**) (**B**), and citromycetin (**3**) (**C**) on the protein expression of iNOS and COX-2 in murine peritoneal macrophages stimulated with LPS. Murine peritoneal macrophages were pre-treated for 12 h at the indicated compound concentrations and stimulated for 18 h with LPS (1 μg/mL). Western blotting analysis was performed, and the representative blots of three independent experiments are shown.

**Figure 4:**
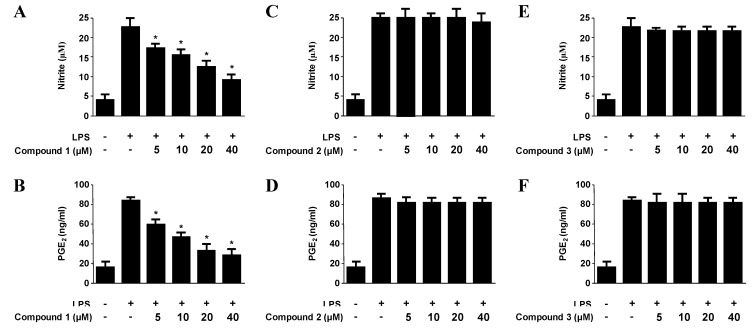
The effects of penstyrylpyrone (**1**), anhydrofulvic acid (**2**), and citromycetin (**3**) on nitrite (**A**, **C**, **E**)and PGE_2_ (**B**, **D**, **F**) production in murine peritoneal macrophages stimulated with LPS. Murine peritoneal macrophages were pre-treated for 12 h at the indicated compound concentrations and stimulated for 18 h with LPS (1 μg/mL). The concentrations of nitrite and PGE_2_ were determined. The data represent the mean values ± SD of three experiments. * *p* < 0.05 compared with the group treated with LPS.

The expression of pro-inflammatory enzymes, including COX-2 and iNOS, plays an important role in immune-activated macrophages via the production of COX-2-derived PGE_2_ and iNOS-derived NO [20,21]. To investigate the effects of compounds on iNOS-derived NO and COX-2-derived PGE_2_ production, and iNOS and COX-2 expression in LPS-stimulated macrophages, murine peritoneal macrophages were stimulated with LPS (1 μg/mL) for 18 h in the presence or absence of non-cytotoxic concentrations of compounds **1**–**3 **(Figure S1). Among the tested compounds, penstyrylpyrone (**1**) exhibited NO and PGE_2_ production in a dose-dependent manner with IC_50_ values of 12.32 μM and 9.35 μM (Table 3), respectively, while anhydrofulvic acid (**2**) and citromycetin (**3**) did not show any reduction of NO and PGE_2_ up to 40 μM. In addition, pre-treatment of the macrophages with penstyrylpyrone (**1**) for 12 h resulted in decreased iNOS and COX-2 expression (Figure 3A), and attenuated the production of iNOS-derived NO and COX-derived PGE_2_ production (Figure 4A). The finding that penstyrylpyrone (**1**) suppressed LPS-induced pro-inflammatory mediators, such as NO, PGE_2_, iNOS, and COX-2, led further investigation of the effects of penstyrylpyrone (**1**) on LPS-induced TNF-α and IL-1β production, which are other pro-inflammatory mediators. When murine peritoneal macrophages were pre-treated with penstyrylpyrone (**1**) for 12 h and subsequently stimulated with LPS, penstyrylpyrone (**1**) was shown to decrease IL-1β (Figure S2A) and TNF-α (Figure S2B) production in a concentration-dependent manner as determined by enzyme immunoassays.

**Table 3:** Effects of **1**–**3** on the NO, PGE_2_, TNF-α, and IL-1β production with IC_50_ values.

Compounds	NO production	PGE_2_ production	IL-1β production	TNF-α production
Penstyrylpyrone (**1**)	12.32 μM	9.35 μM	13.54 μM	18.32 μM
Anhydrofulvic acid (**2**)	>40 μM	>40 μM	>40 μM	>40 μM
Citromycetin (**3**)	>40 μM	>40 μM	>40 μM	>40 μM

### 2.4. Effects of Penstyrylpyrone (1) on the Protein Expression Levels of IκB-α Phosphorylation and Degradation as well as NF-κB Translocation and DNA Binding Activity in Murine Peritoneal Macrophages

**Figure 5:**
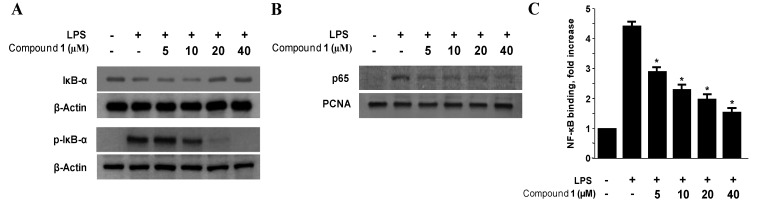
The effects of penstyrylpyrone (**1**) on the protein expression level of IκB-α phosphorylation, degradation of IκB-α (**A**), NF-κB translocation (**B**), and NF-κB DNA binding activity (**C**) in murine peritoneal macrophages. Murine peritoneal macrophages were pre-treated for 12 h at the indicated concentrations of penstyrylpyrone (**1**), and stimulated for 1 h with LPS (1 μg/mL). Western blotting analysis of IκB-α and p-IκB-α in the cytoplasm and NF-κB in the nucleus (**A**, **B**) were performed. A commercially available NF-κB ELISA (Active Motif) was used to test the nuclear extracts and to determine the degree of NF-κB binding (**C**). The data represent the mean values ± SD of three experiments. *p < 0.05 compared with the group treated with LPS.

The transcription factor NF-κB (of which, the p50/p65 heterodimer is the most common) has been implicated in the regulation of many genes that encode for mediators of immune, acute-phase, and inflammatory responses, including iNOS and COX-2 [32]. Under basal conditions, NF-κB is sequestered in the cytoplasm by inhibitor proteins, including IκB; however, when it is released upon stimulation, NF-κB dimers translocate into the nucleus to activate target genes via high affinity binding to κB elements in their promoters [33]. Therefore, the phosphorylation and degradation of IκB-α, an inhibitor of NF-κB nuclear translocation, were evaluated as a next step to determine the mechanisms by which penstyrylpyrone (**1**) suppressed the production of LPS-induced pro-inflammatory enzymes and mediators. As shown in Figure 5, IκB-α in murine peritoneal macrophages was degraded after LPS treatment (1 μg/mL for 1 h). However, pre-treatment of penstyrylpyrone (**1**) for 12 h, at concentrations ranging from 5 to 40 μM, markedly inhibited LPS-induced phosphorylation and degradation of IκB-α, thereby preventing NF-κB (p65) translocation into the nucleus. Nuclear p65 protein levels increased after treatment with LPS for 1 h. However, this response was gradually inhibited by the treatment with penstyrylpyrone (**1**) in a dose-dependent manner. We also observed an increase in the DNA-binding activity of NF-κB in nuclear extracts obtained from murine peritoneal macrophages that were stimulated with LPS for 1 h. Compared with controls, treatment with LPS increased NF-κB DNA-binding activity by approximately 3-fold. However, penstyrylpyrone (**1**) impaired this activity in a concentration-dependent manner (Figure 5C). 

### 2.5. Effects of Penstyrylpyrone (1) on HO-1 Expression via Nuclear Translocation of Nrf2 in Murine Peritoneal Macrophages

**Figure 6:**
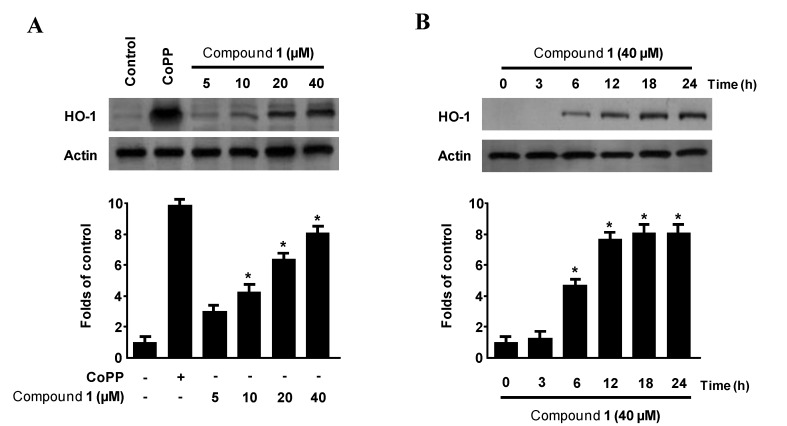
The effects of penstyrylpyrone (**1**) on the protein expression of HO-1 in murine peritoneal macrophages. Murine peritoneal macrophages were incubated for 12 h with the indicated concentrations of penstyrylpyrone (**1**). Western blotting analyses revealed concentration-dependent (**A**) and time-dependent (**B**) HO-1 protein expression. Representative blots of three independent experiments are shown. The data represent the mean ± SD of three experiments. * *p* < 0.05 compared with the control group.

HO-1 has been recognized for its major immunomodulatory and anti-inflammatory properties in a number of inflammation models [16,17]. The anti-inflammatory action of HO-1 is mediated by the inhibition of the production of pro-inflammatory cytokines and chemokines, such as NO, PGE_2_, TNF-α, IL-1β, and IL-6, through NF-κB pathways in activated macrophages [18,19,20,21,34]. Therefore, we next examined whether penstyrylpyrone (**1**) induced HO-1 expression in murine peritoneal macrophages. In cells treated with non-cytotoxic concentrations of penstyrylpyrone (**1**) (5–40 μM for 12 h), we found a concentration-dependent increase in the protein HO-1 expression (Figure 6A). Treatment with penstyrylpyrone (**1**) concentrations above 10 μM showed a considerable increase in HO-1 expression. In addition, treatment with penstyrylpyrone (**1**) concentrations of 40 μM resulted in a temporal increase in HO-1 expression. As shown in Figure 6B, HO-1 protein expression began to increase 6 h after penstyrylpyrone (**1**) treatment, and the maximum level of HO-1 protein expression was reached at 18 h after treatment.

**Figure 7:**
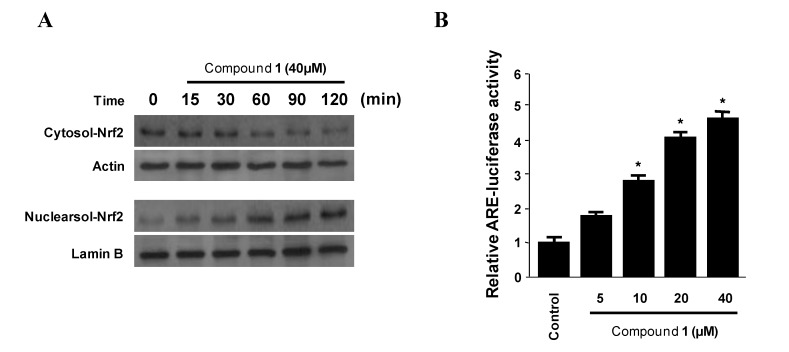
The effects of penstyrylpyrone (**1**) on the nuclear translocation of Nrf2 (**A**) and anti-oxidant response element (ARE) activation (**B**) in murine peritoneal macrophages. Murine peritoneal macrophages were treated with 40 μM of penstyrylpyrone (**1**) for 15, 30, 60, 90 and 120 min. The nuclei were fractionated from the cytosol using PER-Mammalian Protein Extraction buffer. Nrf2 protein was detected using Western blotting analysis and representative blots of three independent experiments are shown. Quiescent cells transiently transfected with ARE-luciferase or control vector were incubated for 1 h with indicated concentrations of latifolin in the presence of 5% FBS. Cell lysates were assayed for luciferase activity and depicted by fold induction by normalizing the transfection efficiency and dividing the values of each experiment relative to the control. * *p* < 0.05 compared to the control group.

The redox-dependent transcription factors such as Nrf2, which is a master regulator of the NF-κB signaling, have been shown to mediate HO-1 induction [22]. In addition, Nrf2, interact with the anti-oxidant response element (ARE), which is the major *cis*-acting regulatory DNA element in the HO-1 gene promoter, the interplay of these two nuclear factor appears to be of major importance for inducible HO-1 gene expression [23]. Thus, we investigated whether the treatment of murine peritoneal macrophages with penstyrylpyrone (**1**) induced the nuclear translocation of Nrf2 and activation of ARE. When macrophages were incubated with penstyrylpyrone (**1**) for 15, 30, 60, 90, and 120 min at a concentration of 40 μM, the nuclear fractions of the macrophages showed a gradual increase in Nrf2 levels with a concomitant decrease in cytoplasmic Nrf2 levels (Figure 7A). In addition, we found that ARE activation gradually increased in a dose-dependent manner (Figure 7B). These suggest that penstyrylpyrone (**1**) was associated with HO-1 expression via Nrf2-ARE pathways.

### 2.6. Effects of SnPP on the Inhibition of Pro-Inflammatory Mediator Production via the Pre-Treatment of Penstyrylpyrone (**1**) in LPS-Stimulated Murine Peritoneal Macrophages

**Figure 8:**
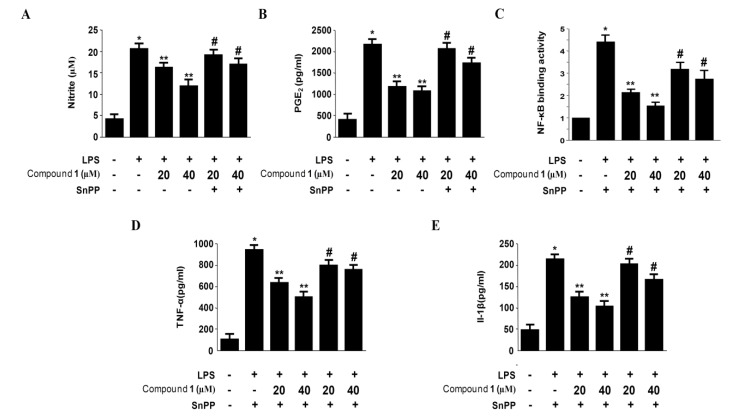
The effects of SnPP on the inhibition of nitrite production (**A**), PGE_2_ production (**B**), NF-κB DNA-binding activity (**C**), TNF-α production (**D**), and IL-1β production (**E**) by penstyrylpyrone (**1**) pre-treatment of LPS-stimulated murine peritoneal macrophages. Murine peritoneal macrophages were pre-treated for 12 h with penstyrylpyrone (**1**) (40μM), in the presence or absence of SnPP (50 μM) and stimulated for 18 h with LPS (1μg/mL). The nitrite (**A**), PGE_2_ (**B**), TNF-α (**D**), and IL-1β (**E**) concentrations and nuclear NF-κB DNA-binding activity (**C**) were investigated as described in Materials and Methods. The data represent the mean ± SD of three experiments. * *p* < 0.05 compared with the control group; ** *p* < 0.05 compared with the group treated with LPS alone; ^#^ *p* < 0.05 compared with the group treated with penstyrylpyrone (**1**) and LPS.

The anti-inflammatory action of HO-1 is mediated by the inhibition of pro-inflammatory cytokines and chemokines production through NF-κB pathways in activated macrophages [16,17,18,19,20]. Thus, it is well known that one of the key regulators of NF-κB signaling is Nrf2-medicated HO-1 expression [21,22,23]. To confirm that the inhibitory effect of penstyrylpyrone (**1**) on the NF-κB signaling pathway (Figure 3, Figure 4, Figure 5) is medicated with the expression of HO-1 via Nrf2 pathway (Figure 6, Figure 7), we examined whether the induction of HO-1 expression by penstyrylpyrone (**1**) was altered by the pre-treatment of SnPP, which is an inhibitor of HO-1 (Figure 8). Murine peritoneal macrophages were pre-treated with 40 μM of penstyrylpyrone (**1**) for 12 h in the absence or presence of SnPP. The suppressive effects of penstyrylpyrone (**1**) on LPS-stimulated NO, PGE_2_, TNF-α, and IL-1β production and NF-κB DNA-binding activity were partially reversed by SnPP (Figure 8). Therefore, it was supported that the induction of HO-1 by penstyrylpyrone (**1**) contributes to the inhibitory effects on the production of pro-inflammatory mediators via NF-κB pathways.

## 3. Experimental Section

### 3.1. General Experimental Procedures and Materials

UV spectra were recorded on a Biochrom 1300 UV/visible spectrophotometer. IR spectra were obtained on a Spectrum GX FT-IR Spectrometer (Perkin Elmer, Hanover, MD, USA). ESIMS data were obtained using a Q-TOF micro LC-MS/MS instrument (Waters, Milford, MA, USA). NMR spectra (1D and 2D) were recorded in DMSO by using a JEOL JNM ECP-400 spectrometer (400 MHz for ^1^H and 100 MHz for ^13^C), and chemical shifts were referenced relative to the residual solvent peaks (δ_H_/δ_C_ = 2.50/39.5 ppm). HSQC and HMBC spectroscopy experiments were optimized for ^1^*J*_CH_ = 140 Hz and ^n^*J*_CH_ = 8 Hz, respectively. Solvents for extraction and open-column chromatography were reagent grade and used without further purification. Solvents used for HPLC were analytical grade. Flash column chromatography was performed using Aldrich octadecyl-functionalized silica gel (C_18_). HPLC separations were performed on a Shiseido Capcell Pak C_18_ column (20 × 150 mm; 5 μm particle size) with a flow rate of 5 mL/min. Compounds were detected by UV absorption at 254 nm.

Dulbecco’s modified Eagle’s medium (DMEM), fetal bovine serum (FBS), and other tissue culture reagents were purchased from Gibco BRL Co. (Grand Island, NY, USA). Tin protoporphyrin IX (SnPP IX), an inhibitor of HO-1, was obtained from Porphyrin Products (Logan, UT, USA). TG was purchased from BD Pharmingen (San Diego, CA, USA). All of the other chemicals were obtained from Sigma Chemical Co. (St. Louis, MO, USA) unless stated otherwise. Primary antibodies, including those raised against HO-1, COX-2, iNOS, IκB-α, p-IκB-α, and p65, and the appropriate secondary antibodies used for western blotting analysis were purchased from Santa Cruz Biotechnology (Santa Cruz, CA, USA). Enzyme-linked immunosorbent assay (ELISA) kits for PGE_2_, TNF-α, and IL-1β were purchased from R & D Systems (Minneapolis, MN, USA).

### 3.2. Specimen Collection and Identification of the Marine-Derived Fungus *Penicillium* sp. JF-55

The fungal strain *Penicillium* sp. JF-55 was isolated from an unidentified sponge that was manually collected using scuba equipment off the shores of Jeju Island in February 2009. The sample was stored in a sterile plastic bag and transported to the laboratory, where it was kept frozen until further processing. The sample was diluted 10 times by using sterile seawater. One milliliter of the diluted sample was processed using the spread plate method in potato dextrose agar (PDA) medium (containing 3% NaCl) plates. The plate was incubated at 25 °C for 14 days. After the isolates were purified several times, the final pure cultures were selected and preserved at −70 °C. This fungus was identified on the basis of an analysis of ribosomal RNA (rRNA) sequences. A GenBank search using the 28S rRNA gene of JF-55 (Genbank accession number JQ342168) indicated *Penicillium glabrum* (AB470560), *Eupenicillium* sp. (FJ800556), and *Chromocleista malachitea* (FJ358281) as the closest matches, which showed sequence identities of 99%, 96%, and 95%, respectively. Thus, the marine-derived fungal strain JF-55 was characterized as *Penicillum* sp. 

### 3.3. Isolation of Compounds **1**–**3** from the Marine-Derived Fungus *Penicillium* sp. JF-55

The fungal strain was cultured on 50 petri-dish plates (90 mm), each containing 20 mL of PDA media (0.4% (w/v) potato starch, 2% (w/v) dextrose, 3% (w/v) NaCl, 1.5% (w/v) agar). The plates were individually inoculated with 20 mL of the fungal strain culture and incubated at 25 °C for a period of 14 days. Extraction of the agar media by using methylethylketone (2 × 500 mL) provided an organic phase, which was then concentrated *in vacuo* to yield 878.6 mg of the extract. The extract was subjected to C_18_ flash column chromatography (5 × 40 cm), and eluted using a stepwise gradient of 20%, 40%, 60%, 80%, and 100% (v/v) MeOH in H_2_O (500 mL each). The eluted fractions at 80% MeOH (66.8 mg) were purified using semi-preparative reversed-phase HPLC elution with a gradient from 40% to 80% MeOH in H_2_O (0.1% formic acid) over 50 min to yield compound **1** (13.8 mg, *t*_R_ = 24.3 min) and compound **2** (6.9 mg, *t*_R_ = 19.6 min). A portion (52.5 mg) of the eluted fractions at 40% MeOH (201.1 mg) was purified using semi-preparative reversed-phase HPLC elution with a gradient from 20% to 80% MeOH in H_2_O (0.1% formic acid) over 60 min to yield compound **3** (19.8 mg, *t*_R_ = 26.0 min).

Data for penstyrylpyrone (**1**): dark brown gum; UV (MeOH) λ_max_ nm (log ε): 212(4.13), 236(4.07), 363(4.15); IR (KBr) ν_max_ 3443, 3083, 2950, 1688, 1551, 1465, 1378, 1268, 1144, 1018, 969, 750, 690 cm^−1^; ^1^H NMR and ^13^C NMR data, see Table 1; HRESIMS *m*/*z* 243.1033 [M + H]^+^ (calculated for C_15_H_15_O_3_, 243.1021).

### 3.4. PTP1B Assay

PTP1B (human, recombinant) was purchased from BIOMOL Research Laboratories, Inc. Enzyme activity was measured in a reaction mixture containing 2 mM *p*-nitrophenyl phosphate (*p*NPP) in 50 mM citrate, pH 6.0 (composition: 0.1 M NaCl, 1 mM EDTA, and 1 mM dithiothreitol (DTT)). The reaction mixture was placed in an incubator maintained at 30 °C for 30 min, and the reaction was terminated by the addition of 1 N NaOH. The amount of *p*-nitrophenol produced was estimated by measuring the increase in absorbance at 405 nm. The nonenzymatic hydrolysis of 2 mM *p*NPP was corrected by measuring the increase in absorbance at 405 nm in the absence of PTP1B enzyme [35,36]. For the kinetic analysis, the reaction mixture consisted of different concentrations of *p*NPP as a PTP1B substrate in the absence or presence of compound **1**, and assays were performed as described above. The Michaelis-Menten constant (*K*_m_) and maximum velocity (*V*_max_) of PTP1B were determined by the Lineweaver-Burk plot using a GraphPad Prism^®^ 4 program (GraphPad Software Inc., San Diego, CA, USA).

### 3.5. Peritoneal Macrophage Cultures

C57BL/6 mice were purchased from Orient Bio Co. (Sungnam, Kyung-Kido, Korea). TG-elicited peritoneal macrophages were harvested four days after intraperitoneal (i.p.) injection of 3 mL of TG [37]. Peritoneal lavage was performed using 8 mL of Hanks’ Balanced Salt Solution containing 10 U/mL heparin. The cells were distributed in Roswell Park Memorial Institute medium (RPMI) supplemented with 10% heat-inactivated FBS, in 6-well tissue culture plates (5 × 10^6^ cells/mL).

### 3.6. Cell Viability Assay

The effects of various experimental modulations on cell viability were evaluated according to mitochondrial reductase function by using an assay based on the reduction of tetrazolium salt 3-[4,5-dimethylthiazol-2-yl]-2,5-diphenyltetrazolium bromide (MTT) into formazan crystals [38].

### 3.7. Determination of Nitrite Production

The production of nitrite, a stable end product of NO oxidation, was used as a measure of iNOS activity. The nitrite present in the conditioned medium was determined using a method based on the Griess reaction [39].

### 3.8. PGE_2_, TNF-α and IL-1β Assay

The level of PGE_2_, TNF-α or IL-1β present in each sample was determined using a commercially available kit from R & D Systems [40]. The assay was performed according to the manufacturer’s instructions. Briefly, murine peritoneal macrophages were cultured in 24-well plates, pre-incubated for 12 h with different concentrations of compounds **1**–**3**, and then stimulated for 18 h with LPS. The cell culture supernatants were then immediately collected after treatment and centrifuged at 13,000× *g* for 2 min to remove particulate matter. The medium was added to a 96-well plate pre-coated with affinity-purified PGE_2_-specific polyclonal antibodies or the medium was added to a 96-well plate pre-coated with affinity-purified polyclonal antibodies that were specific to mouse TNF-α or IL-1β. An enzyme-linked polyclonal antibody specific for PGE_2_, mouse TNF-α or IL-1β was added to the wells for 20 h, followed by a final wash to remove any unbound antibody-enzyme reagent. A substrate solution was added, and the intensity of the color produced, which was measured at 450 nm (the correction wavelength was set at 540 nm or 570 nm), was proportional to the amount of PGE_2_, TNF-α or IL-1β present.

### 3.9. Preparation of Cytosolic and Nuclear Fractions

Murine peritoneal macrophages were homogenized (1:20, w:v) in PER-Mammalian Protein Extraction buffer (Pierce Biotechnology, Rockford, IL, USA) containing freshly added protease inhibitor cocktail I (EMD Biosciences, San Diego, CA, USA) and 1 mM phenylmethylsulfonyl fluoride (PMSF). The cytosolic fraction of the cells was prepared by centrifugation at 15,000× *g* for 10 min at 4 °C. Nuclear and cytoplasmic extracts of murine peritoneal macrophages were prepared using NE-PER nuclear and cytoplasmic extraction reagents (Pierce Biotechnology). After treatment, the murine peritoneal macrophages (3 × 10^6^ cells/3 mL in 60 mm dishes) were collected and washed with phosphate-buffered saline (PBS). After centrifugation, cell lysis was performed at 4 °C by vigorous shaking for 15 min in a radioimmunoprecipitation assay (RIPA) buffer (150 mM NaCl, 1% NP-40, 0.5% sodium deoxycholate, 0.1% SDS, 50 mM Tris-HCl (pH 7.4), 50 mM glycerophosphate, 20 mM NaF, 20 mM ethylene glycol tetraacetic acid (EGTA), 1 mM dithiothreitol (DTT), 1 mM Na_3_VO_4_, and protease inhibitors). After centrifugation at 15,000× *g* for 15 min, the supernatant was separated and stored at −70 °C until further use. The protein concentration was determined using the bicinchoninic acid (BCA) protein assay kit.

### 3.10. Western Blotting Analysis

Western blotting analysis was performed by lysing the cells in 20 mM Tris-HCl buffer (pH 7.4) containing a protease inhibitor mixture (0.1 mM PMSF, 5 mg/mL aprotinin, 5 mg/mL pepstatin A, and 1 mg/mL chymostatin). The protein concentration was determined using a Lowry protein assay kit (P5626; Sigma Chemical Co., St. Louis, MO, USA). An equal amount of protein for each sample was resolved using 12% sodium dodecyl sulfate-polyacrylamide gel electrophoresis (SDS-PAGE) and then electrophoretically transferred onto a Hybond enhanced chemiluminescence (ECL) nitrocellulose membrane (Bio-Rad, Hercules, CA, USA). The membrane was blocked with 5% skimmed milk and sequentially incubated with primary antibody (Santa Cruz Biotechnology, Santa Cruz, CA, USA) and horseradish peroxidase-conjugated secondary antibody followed by ECL detection (Amersham Pharmacia Biotech, Piscataway, NJ, USA).

### 3.11. DNA-Binding Activity of NF-κB

The DNA-binding activity of NF-κB in the nuclear extracts was measured using the TransAM kit (Active Motif, Carlsbad, CA, USA) according to the manufacturer’s instructions.

### 3.12. Statistical Analysis

The data were expressed as the mean ± standard deviation (SD) of at least three independent experiments. To compare three or more groups, one-way analysis of variance (ANOVA) followed by the Newman-Keuls *post hoc* test was used. Statistical analysis was performed using GraphPad Prism software, version 3.03 (GraphPad Software Inc., San Diego, CA, USA).

## 4. Conclusions

Chemical investigation of the marine-derived fungus *Penicillium* sp. JF-55 afforded a new styrylpyrone-type metabolite penstyrylpyrone (**1**), along two known metabolites, anhydrofulvic acid (**2**) and citromycetin (**3**). Although phenylpropanoids are well-known plant secondary metabolites, very little is known about phenylpropanoids in the fungal metabolites community [25]. Styrylpyrones such as hispidine are the most abundant phenylpropanoids found in fungi, and these types of compounds are thought to be biosynthesized by the polyketide pathway [41]. In particular, mushrooms are known to produce a variety of styrylpyrones and their biological effects, including anti-oxidative, anti-cancer, anti-platelet, anti-diabetic, anti-inflammatory, and anti-viral activities, have been shown [42]. Anhydrofulvic acid (**2**) and citromycetin (**3**) are members of an under-represented structural class that forms via a single linear heptaketide, and their weak CD4-binding activity has been previously reported [43]. In addition, the anti-fungal activity of anhydrofulvic acid and its mode of action have been previously investigated [28], and citromycetin has been patented for the treatment of neurodegenerative diseases such as Alzheimer’s, Lewy body, and Parkinson’s disease [29]. However, to the best our knowledge, PTP1B inhibitory activity of styrylpyrones and anhydrofulvic acid (**2**) has never been reported. In the course of further pharmacological evaluation of the compounds **1**–**3**, it was also shown that penstyrylpyrone (**1**) suppressed the production of pro-inflammatory mediators such as NO, PGE_2_, TNF-α, and IL-1β via the inhibition of NF-κB pathway probably through the expression of anti-inflammatory HO-1 expression in LPS-induced murine peritoneal macrophages. It is noteworthy that several studies have indicated that the production of pro-inflammatory mediators, such as TNF-α regulate PTP1B overexpression in obesity on the models of *in vivo* and *in vitro* [44,45]. In addition, PTP1B was suggested to be a key contributor to TNF-α-induced insulin resistance and inflammatory conditions in obesity by the other study showing that deficiency of PTP1B ameliorates pro-inflammatory TNF-α-induced insulin resistance and obesity-associated inflammation during aging [45,46]. Therefore, it was suggested that PTP1B is implicated in the development of inflammation and insulin resistance associated with obesity during aging [44,45,46]. Taken together, penstyrylpyrone (**1**) is of interest due to the potential of modulating multiple targets, and may be a potential therapeutic candidate for the treatment of both type II diabetes and inflammatory diseases.

## Supplementary Files

Supplementary File 1 Supplementary Information (PDF, 1167 KB)
