# Supplementary material for: PTP1B Inhibitory and Anti-Inflammatory Effects of Secondary Metabolites Isolated from the Marine-Derived Fungus Penicillium sp. JF-55
Source: Mar Drugs. 2013 Apr 23;11(4):1409–26. doi: 10.3390/md11041409 (PMC3705412; doi:10.3390/md11041409)

Supplementary Information

Table of Contents

Figure S1. The effects of compounds on cell viability in murine peritoneal macrophages.
Figure S2. The effects of penstyrylpyrone (**1**) on IL-1β and TNF-α production in murine peritoneal macrophages stimulated with LPS.
Figure S3. ^{1}H NMR spectrum of penistyrylpyrone (**1**) in DMSO-d_6.
Figure S4. ^{13}C NMR spectrum of penistyrylpyrone (**1**) in DMSO-d_6.
Figure S5. HSQC data of penistyrylpyrone (**1**) in DMSO-d_6.
Figure S6. HMBC data of penistyrylpyrone (**1**) in DMSO-d_6.
Figure S7. ^{1}H NMR spectrum of anhydrofulvic acid (**2**) in DMSO-d_6.
Figure S8. ^{13}C NMR spectrum of anhydrofulvic acid (**2**) in DMSO-d_6.
Figure S9. ^{1}H NMR spectrum of citromycetin (**3**) in DMSO-d_6.
Figure S10. ^{13}C NMR spectrum of citromycetin (**3**) in DMSO-d_6.

Figure S1. The effects of compounds on cell viability in murine peritoneal macrophages. Murine peritoneal macrophages were incubated for 48 h with various concentrations of each compound (1–50 μM). Cell viability was determined. The data represent the mean values ± SD of 3 experiments.

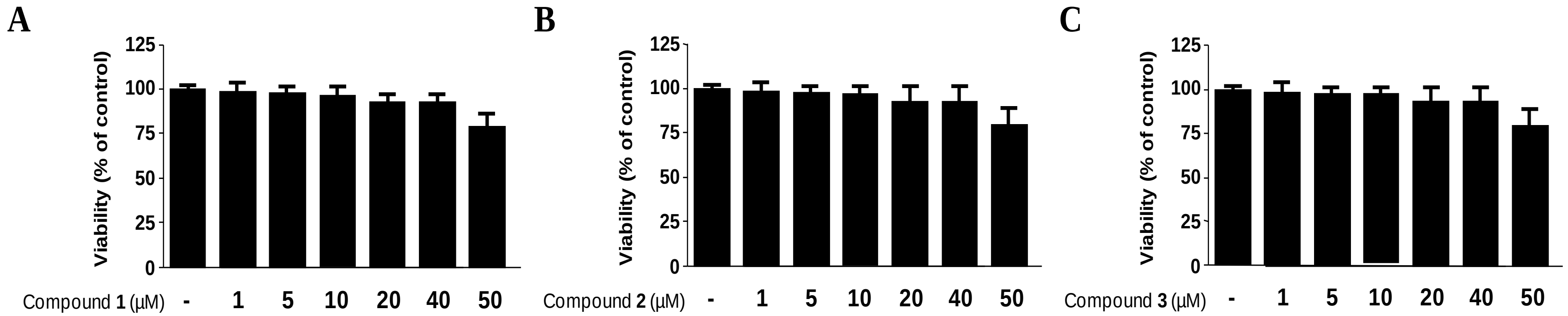

Figure S2. The effects of penstyrylpyrone (**1**) on IL-1β (**A**) and TNF-α (**B**) production in murine peritoneal macrophages stimulated with LPS. Murine peritoneal macrophages were pre-treated for 12 h at the indicated concentrations of penstyrylpyrone (**1**) and stimulated for 18 h with LPS (1 μg/mL). The concentrations of IL-1β (**A**) and TNF-α (**B**) were determined. The data represent the mean values ± SD of 3 experiments. * $p < 0.05$ compared with the group treated with LPS.

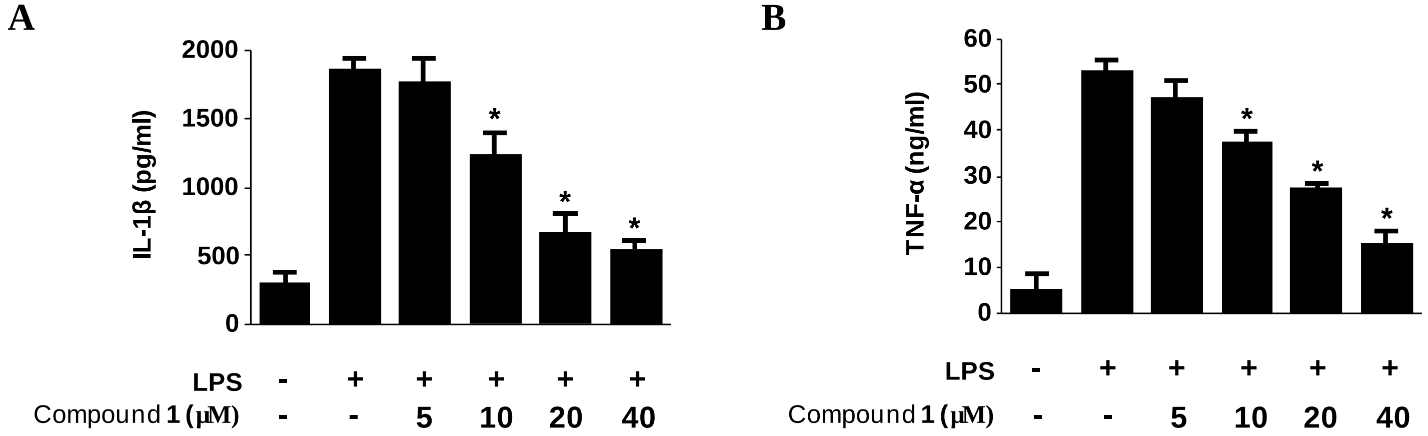

Figure S3. ^{1}H NMR spectrum of penistyrylpyrone (**1**) in DMSO-d_6.

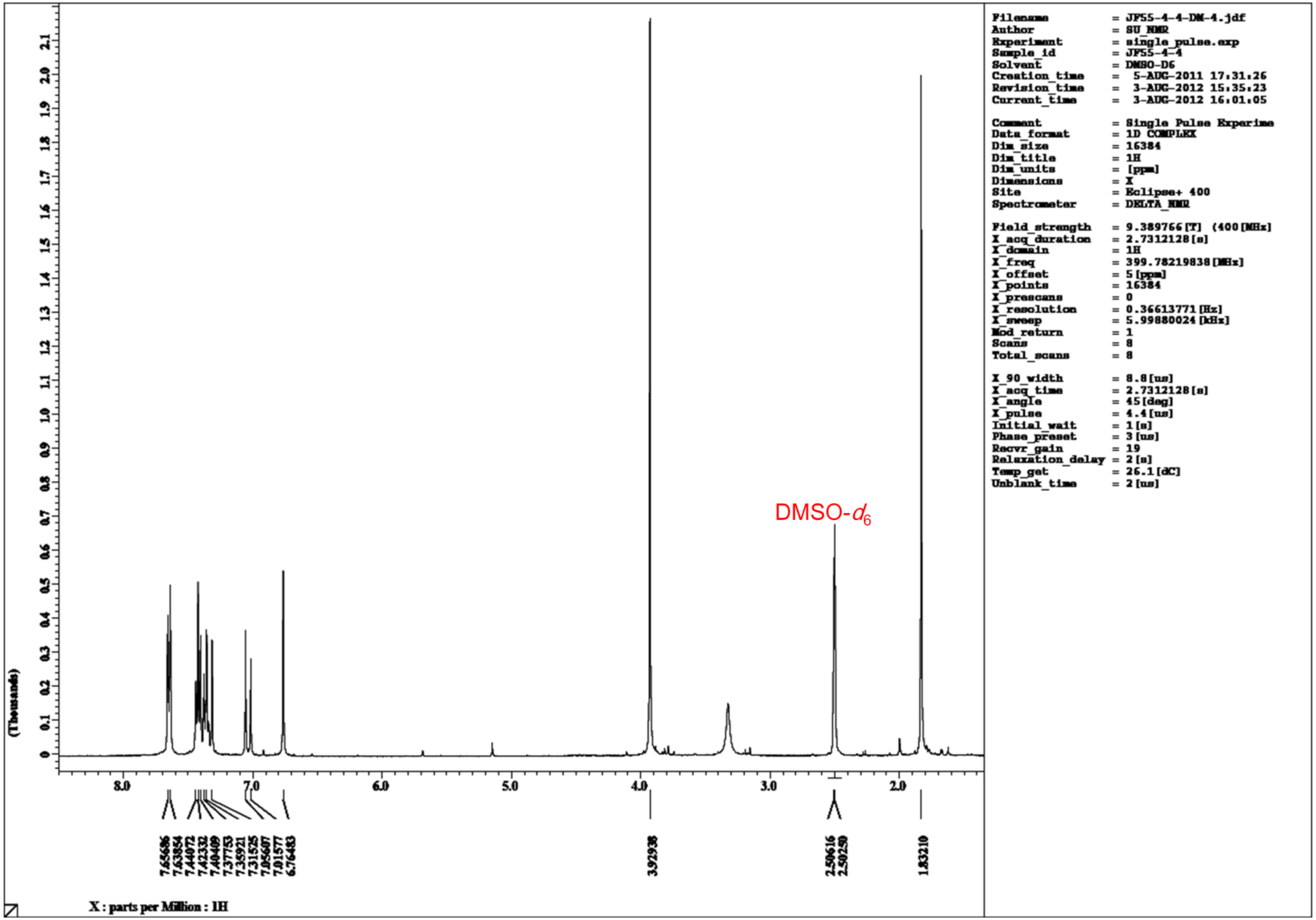

Figure S4. ^{13}C NMR spectrum of penistyrylpyrone (**1**) in DMSO-d_6.

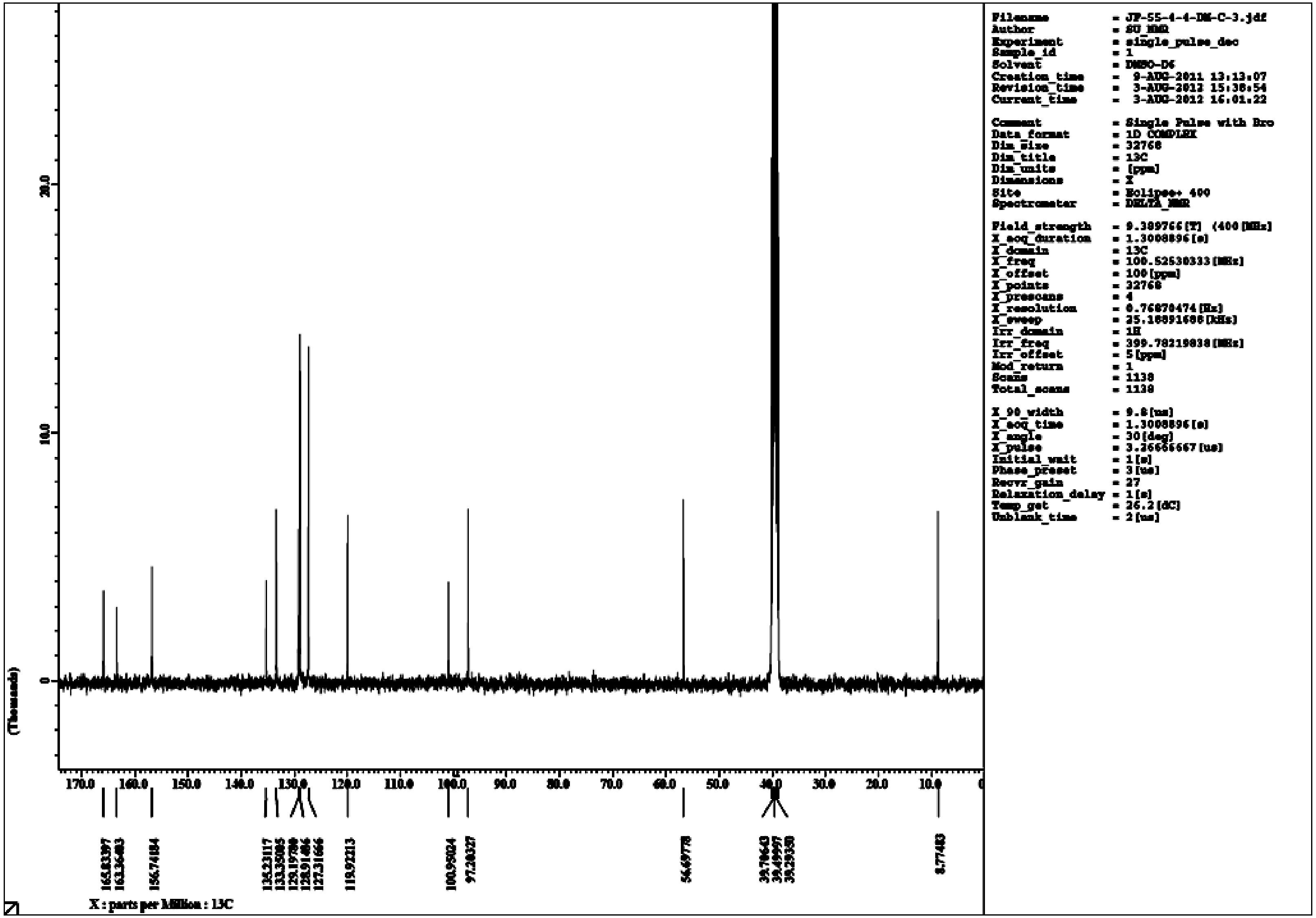

Figure S5. HSQC data of penistyrylpyrone (**1**) in DMSO-d_6.

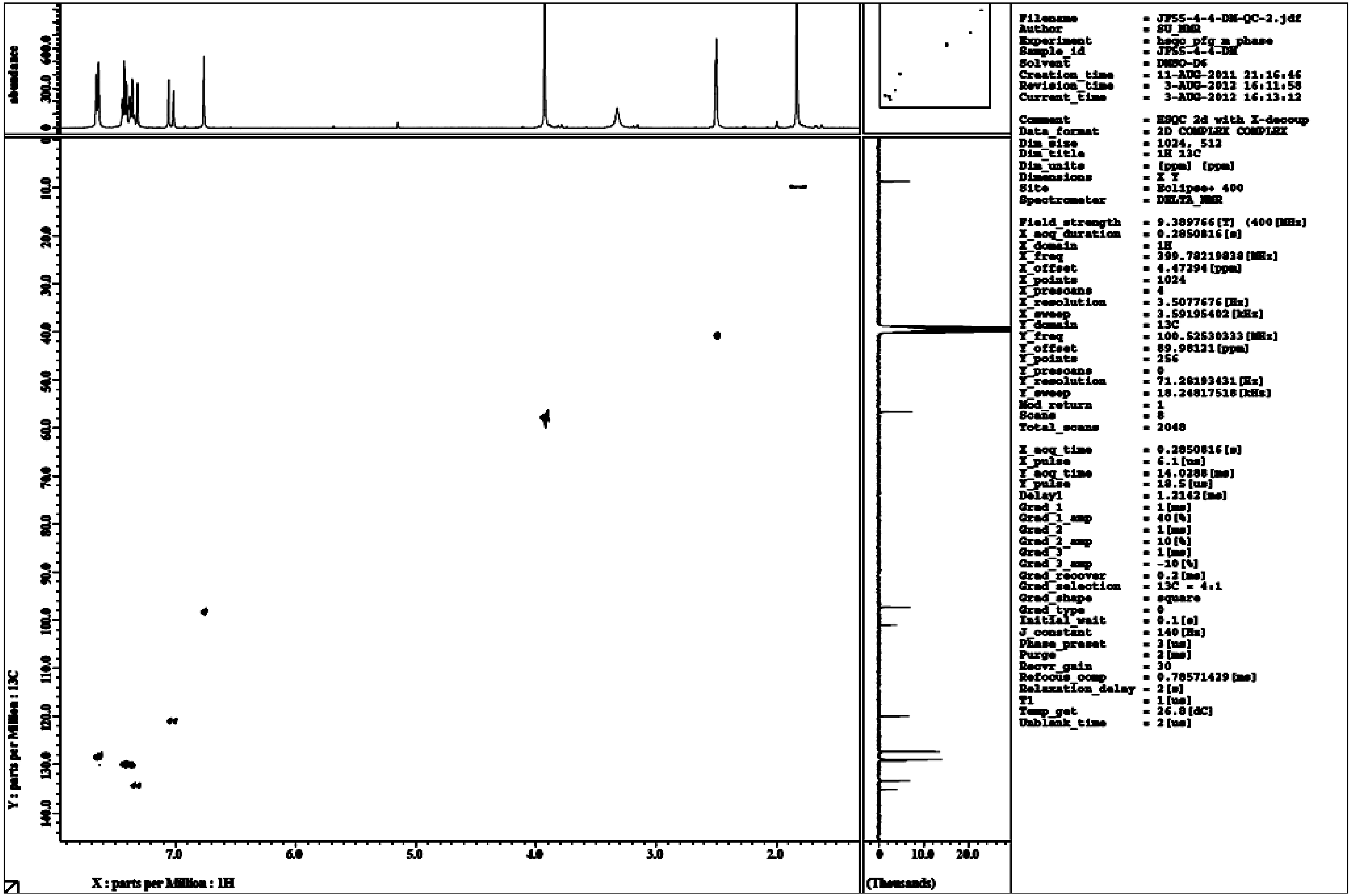

Figure S6. HMBC data of penistyrylpyrone (**1**) in DMSO-d_6.

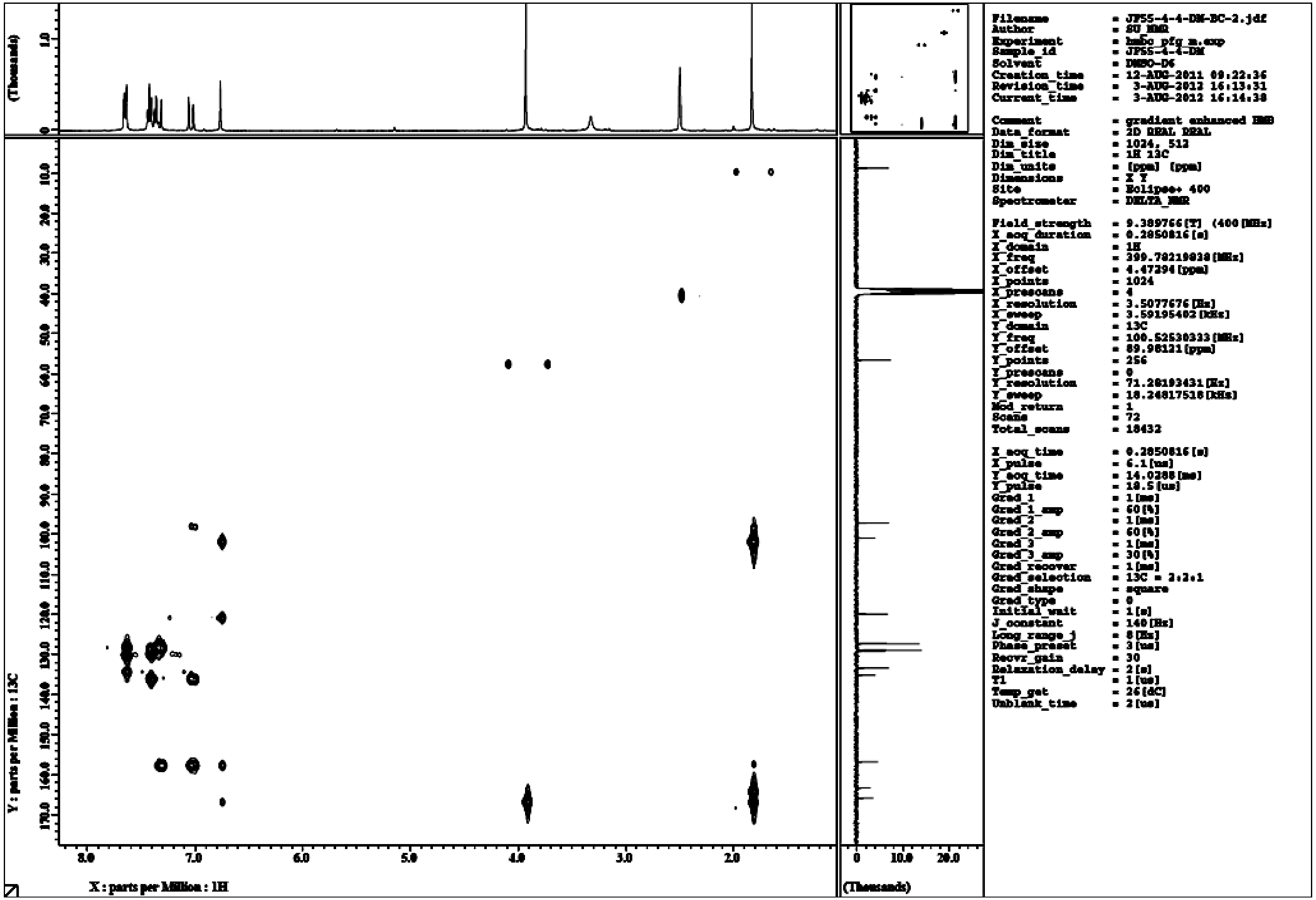

Figure S7. ^{1}H NMR spectrum of anhydrofulvic acid (**2**) in DMSO-d_6.

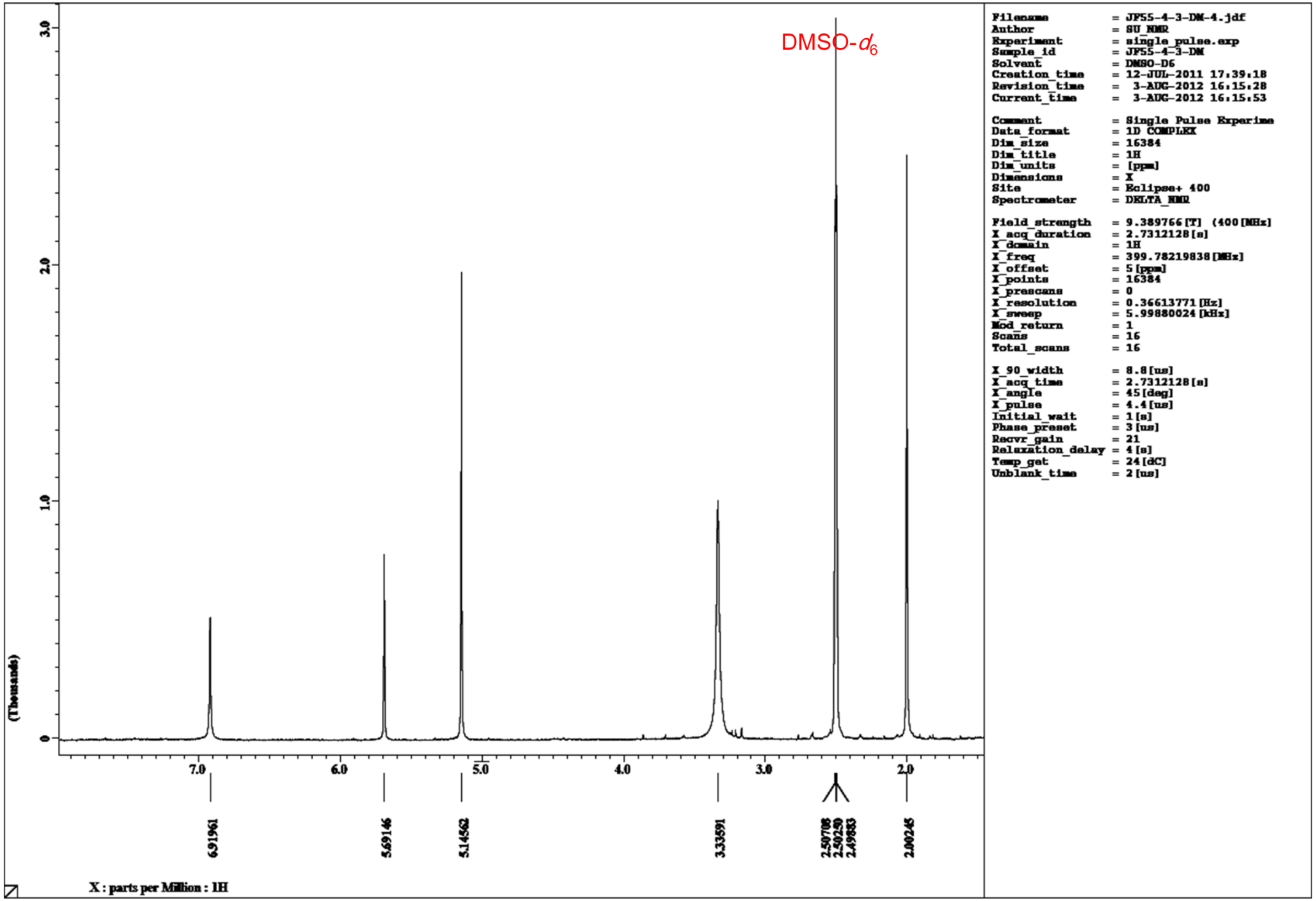

Figure S8. ^{13}C NMR spectrum of anhydrofulvic acid (**2**) in DMSO-d_6.

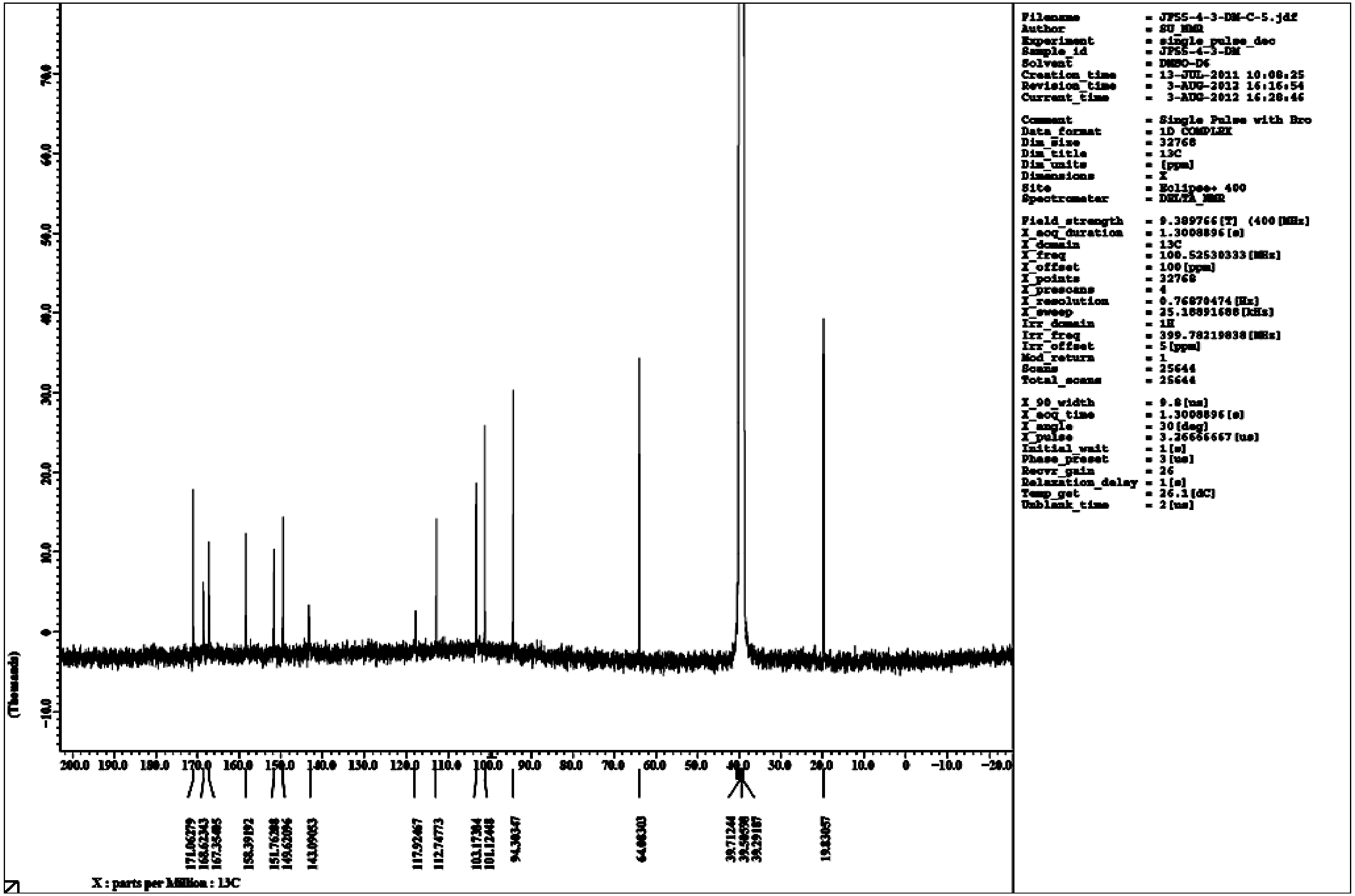

Figure S9. ^{1}H NMR spectrum of citromycetin (**3**) in DMSO-d_6.

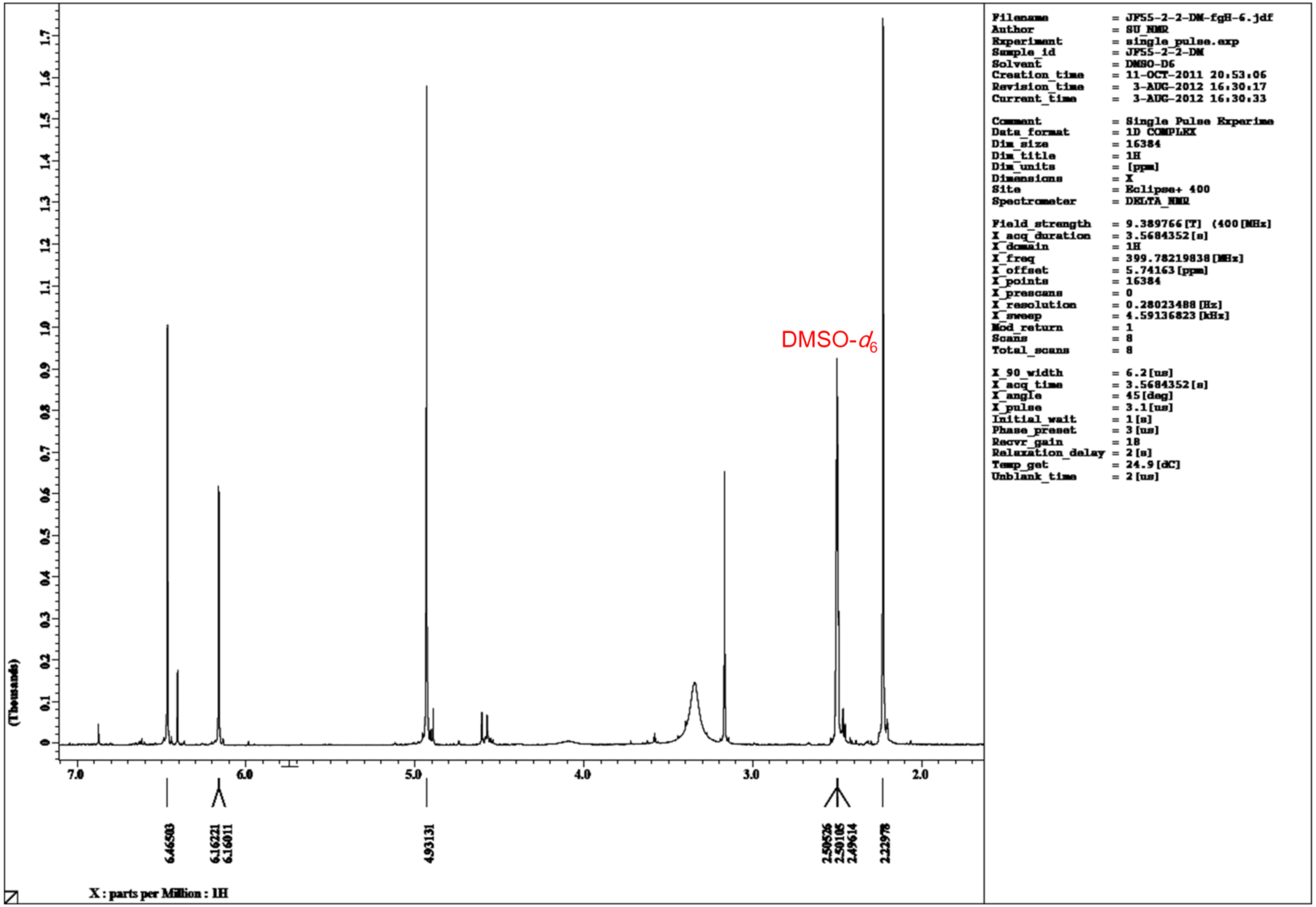

Figure S10. ^{13}C NMR spectrum of citromycetin (**3**) in DMSO-d_6.

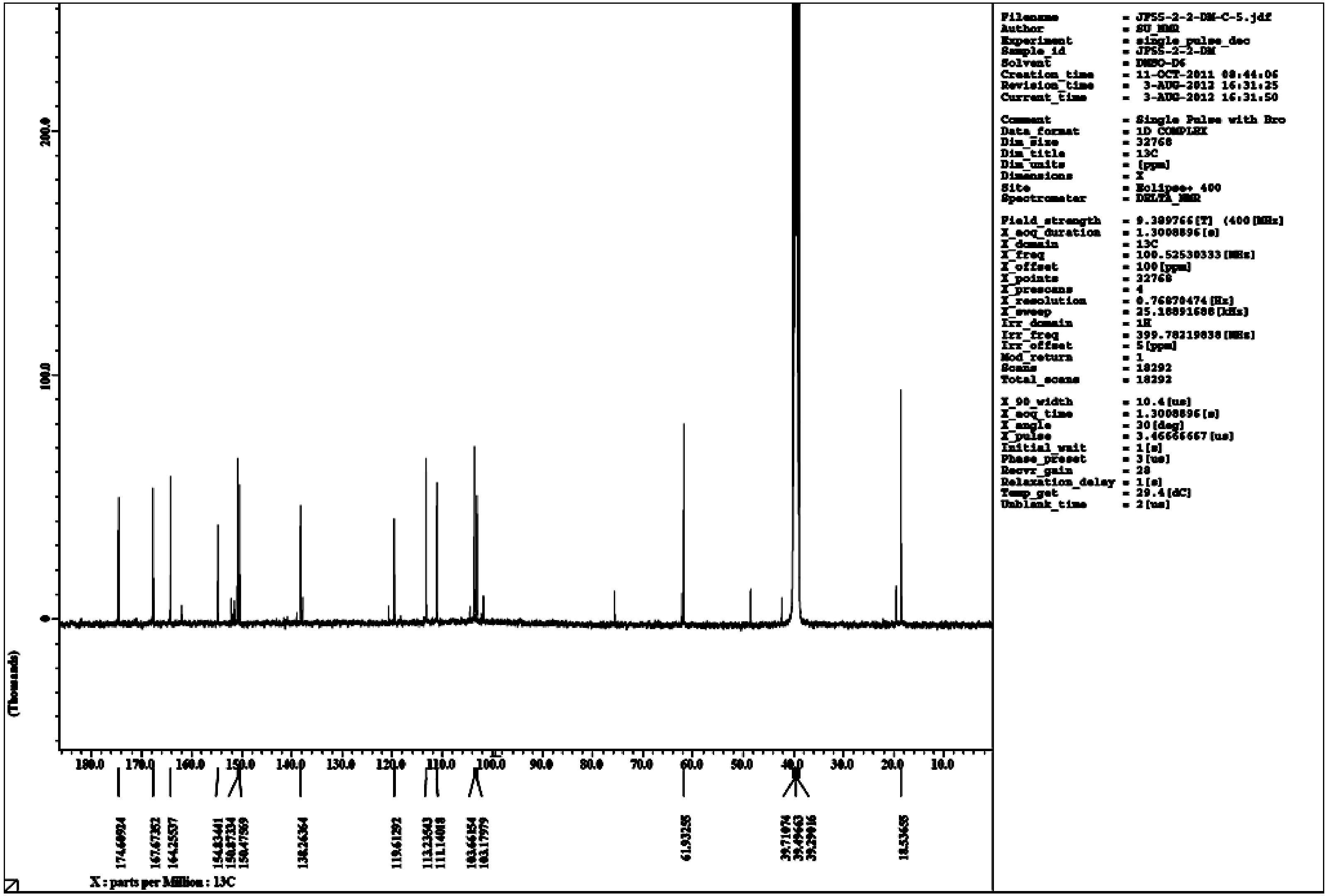

Supplement: Supplementary File 1 — Supplementary Information (PDF, 1167 KB) [file marinedrugs-11-01409-s001.pdf]
